# MENTORED-BASED AGING RESEARCH IN THE BEHAVIORAL AND SOCIAL SCIENCES: RCMAR VI NCC, AN INFRASTRUCTURE FOR SUCCESS

**DOI:** 10.1093/geroni/igae098.0682

**Published:** 2024-12-31

**Authors:** Lisa Barnes

**Affiliations:** Rush University Medical Center, Chicago, Illinois, United States

## Abstract

The RCMAR VI NCC is led by a diverse, multidisciplinary MPI team, with the purpose to develop a robust infrastructure and community that will grow and diversify the research workforce in the behavioral and social sciences to address minority aging, AD/ADRD, and/or health disparities among older adults. This goal will be achieved through the following strategies. 1) Provide support and interaction among RCMAR Centers via an interactive website, robust community-wide online networking platform, tools to enhance collaboration, and resources supporting the creation of new scholarship. 2) Leverage existing and develop new evidence-based tools to provide early career scientists and mentors from diverse backgrounds with skills and technical support necessary to advance their professional growth, foster culturally aware and responsive mentorship and career development. 3) Catalyze resource sharing between and among the RCMAR Centers and cultivate sustainable engagement with key stakeholders including Minority serving institutions; Historically Black Colleges and Universities, Tribal Colleges and Universities, Hispanic Serving Institutions and other academic institutions serving predominately racially minoritized students and groups, all NIA research Centers, the aging research community, and the public. 4) Foster reverence for the value of Diversity, Equity, Inclusion and Anti-Racism (DEIA) in the aging research enterprise by broadly elevating and disseminating the activities and findings of the Centers and their scientists in academic and public venues. 5) Establish consensus on essential common evaluation metrics and implement a streamlined process for collecting data to support the evaluation of RCMAR Center activities, including research, collaboration, mentoring, networking, stakeholder engagement, and DEIA.

